# Criminal Defectors Lead to the Emergence of Cooperation in an Experimental, Adversarial Game

**DOI:** 10.1371/journal.pone.0061458

**Published:** 2013-04-23

**Authors:** Maria R. D'Orsogna, Ryan Kendall, Michael McBride, Martin B. Short

**Affiliations:** 1 Department of Mathematics, California State University, Northridge, Los Angeles, California, United States of America; 2 Department of Economics, University of California Irvine, Irvine, California, United States of America; 3 Department of Mathematics, University of California Los Angeles, Los Angeles, California, United States of America; University of Maribor, Slovenia

## Abstract

While the evolution of cooperation has been widely studied, little attention has been devoted to adversarial settings wherein one actor can directly harm another. Recent theoretical work addresses this issue, introducing an adversarial game in which the emergence of cooperation is heavily reliant on the presence of “Informants,” actors who defect at first-order by harming others, but who cooperate at second-order by punishing other defectors. We experimentally study this adversarial environment in the laboratory with human subjects to test whether Informants are indeed critical for the emergence of cooperation. We find in these experiments that, even more so than predicted by theory, Informants are crucial for the emergence and sustenance of a high cooperation state. A key lesson is that successfully reaching and maintaining a low defection society may require the cultivation of criminals who will also aid in the punishment of others.

## Introduction

The punishment of defectors is vital for sustaining cooperation in social dilemmas [1]–[3]. Indeed, evidence reveals that humans are willing to suffer personal losses in order to punish defectors [4]–[7]. It is thus natural to ask how cooperation and punishment coevolve, as well as if, and how, a society mired in a high incidence of defection can transition to a cooperative state. Several works propose various channels through which this evolution may occur [1], [2], [8]–[14].

Yet, previous game theoretic and experimental research on human cooperation has generally ignored settings with distinctly adversarial characteristics, wherein one actor is uniquely positioned to harm another. This lacuna is puzzling given that such settings better reflect the asymmetries inherent in many social interactions and given the many sociological studies of adversarial behavior within various societies. For example, consider criminal activity, a prototypical adversarial interaction. Criminological research has found that civilians play a crucial role in fostering peaceful communities [15] and self-regulating pro-social norms [16], [17]. Conversely, crime is found to be rampant in disorganized societies where a common understanding of norms and a shared sense of responsibility are absent [18]–[22]. Fear of retaliation against those who cooperate with authorities may be sufficiently strong to undermine the enforcement of pro-social norms; both victim and witness may fear retaliation and disengage from cooperation with law enforcement, leaving criminal behavior to proliferate [23]–[25].

**Figure 1:**
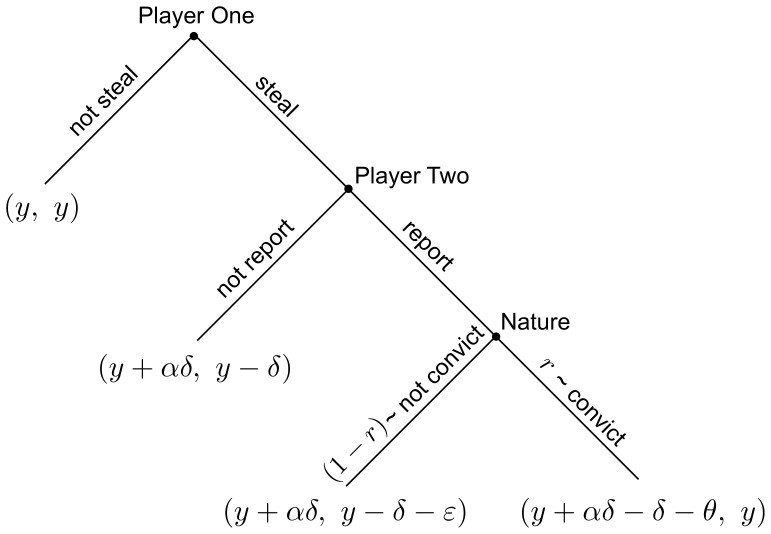
The adversarial interaction represented in a game tree. The final payoffs in the format (player one, player two) are given at the terminal nodes.

Recent theoretical work [26], [27] shifts the focus from standard social dilemmas towards the emergence of cooperation in an adversarial, criminal setting that incorporates the above sociological considerations. The game-theoretic model presented in these works considers two actors selected at random from a large population of 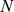 individuals. Each is given an endowment 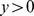. One actor, denoted player one, is placed in a potential criminal role, while the other, denoted player two, is placed in a potential victim role. As depicted in Fig. 1, the former decides whether or not to “steal” an amount 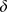 (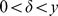) from the other, and the latter decides whether or not to report such theft, if it occurs, to authorities. If the victim does not report, the criminal keeps an amount 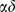 (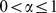), with 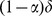 inefficiently lost during the theft. On the other hand, if the victim does report, the criminal is convicted and punished with probability 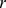, which is equal to the fraction of the total population that would also report under such circumstances; with probability 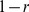, therefore, the criminal is not convicted. Importantly, a convicted criminal fully reimburses the victim's 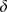 loss and also pays an additional punishment cost 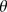, while the victim suffers an additional retaliation cost 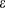 in the case of an unsuccessful reporting. Thus, reporting is a risky proposition for the victim, as a reporting that leads to conviction returns the victim's endowment to 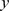, but a reporting that fails to convict leaves the victim worse off than if he or she hadn't reported at all, due to the retaliation cost 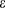. Given the above model, each actor falls into one of four strategies, P, A, I, or V, where
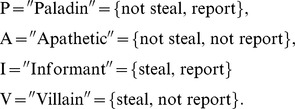
The two-part strategy prescribes what the actor will do for either role in which he or she is placed. Prior works [28], [29] have considered the effects of strategies analogous to the four listed above, but in the framework of public goods games.

**Figure 2:**
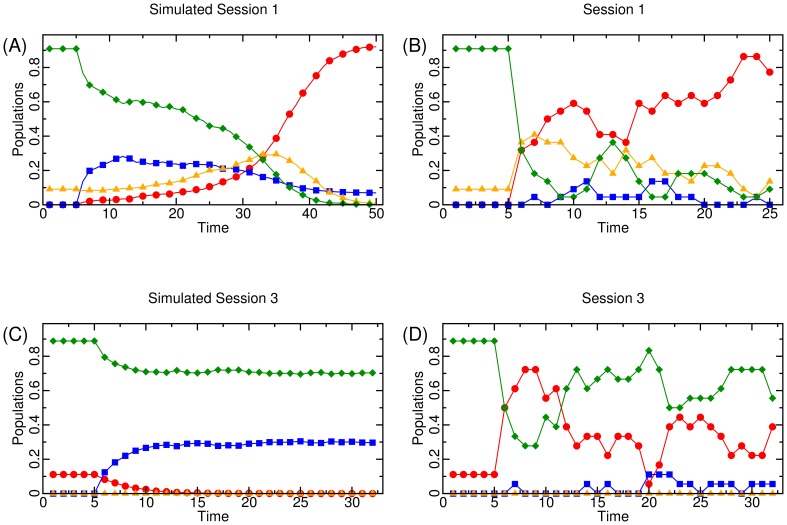
Comparisons of the strategy evolutions for the theoretical imitation dynamic (left figures) and two experimental sessions (right figures). In (A) and (B), all strategies are allowed; in (C) and (D), Informants are disallowed. In all figures, Paladins are red circles, Apathetics are blue squares, Informants are orange triangles, and Villains are green diamonds.

Mathematical analysis and simulations reveal that the Informant strategy (first-order defector, second-order cooperator) plays a critical role in the coevolution of cooperation and punishment when this adversarial interaction is repeated over the course of many periods and actors are given the opportunity to adapt their strategies over time in certain prescribed ways. Two steady states can emerge over such repeated play: high crime, Villain-dominated “Dystopia” or low crime, Paladin-dominated “Utopia”. Under an imitation strategy updating rule [26], the presence of even a small number of Informants within the population is often sufficient to guarantee that the system will end in Utopia, even if the system is initiated very near Dystopia, regardless of game parameters. Figure 2(A) displays results from a simulation using this imitation updating rule, and shows the emergence of cooperation in a society initialized near Dystopia but containing a small number of Informants (90% Villains - 10% Informants). The rise of Informants precedes that of Paladins, which eventually leads to Utopia, a typical pattern replicated in these simulations. In Fig. 2(C), however, the Informant strategy is not allowed and the system does not reach Utopia, despite similar initial conditions (90% Villains - 10% Paladins). Under a best response updating rule [27], a small number of Informants is typically insufficient to cause the system to converge to Utopia, but the *availability* of the Informant strategy is nevertheless a necessary condition for the evolution toward and maintenance of the Utopian state, albeit only under favorable game parameters and initial conditions.

Although the main finding of this prior theoretical work – that Informants are crucial to keeping criminal defection low – closely mirrors anecdotal evidence gathered by law enforcement agencies, there has been no systematic testing involving human subjects to determine the exact role Informants play in promoting cooperative behavior within a society. Our current work aims to shed light on this issue by presenting the results of a series of behavioral science experiments that were designed to mimic as closely as possible the game outlined above, and that specifically address the question of whether and how Informants may foster long-term cooperation.

## Methods

We conducted 16 sessions under 10 different treatments of a computer-based experiment at the Experimental Social Science Laboratory at the University of California, Irvine; the experiment was approved by UC Irvine's Institutional Review Board (IRB) under protocol HS# 2011-8378, with informed consent obtained via the laboratory's website and in-lab instructions. Budgetary constraints forced us to implement a partial factorial over the treatment variables of interest, which include differing initial states, two different parameter profiles, and different combinations of allowed strategies. Students enrolled at the university were contacted via email advertisements to register to be in the laboratory's online subject pool. For each experimental session, an email was sent to all eligible students in the subject pool, and eligible students then signed-up for the session via the laboratory's web site. Each student was only allowed to participate in one of the 16 sessions; that is, a student was eligible if and only if he or she had not yet participated in one of the prior conducted sessions of the experiment. Every session started at 10:00AM and lasted about an hour. Subjects showed up to the lab, signed in, proved their identity by showing their student ID cards, and were assigned a computer terminal. See Table S1 for aggregated information about the subjects who participated in each session.

The experiment itself consists of three phases – instructions, multiple rounds of decision making, and a questionnaire – and was implemented using the z-Tree software package [30]. Our experiment software is meant to imitate the adversarial game described above. The experiment was designed to be free of confounding “visual” variables, such as gender, race, age, etc. Hence, the computer terminals are separated by dividers, which abstract away any identifying features of other participants in the room that might cause a bias upon decision making. The subjects within each session advance through each screen together. That is, the next screen in the experiment is not displayed until all of the subjects in the session have clicked on the appropriate button signaling that they are finished with the current screen.

The instructions inform the subjects about the basic structure of the adversarial setting. They are told that they will be randomly assigned into groups of two and randomly given a role of either “potential criminal” or “potential injured party”. They are also informed about how the points that they earn during the session will be converted into real dollars that they will be payed at the end of the experiment. Finally, they are asked a number of questions to test their understanding of the game and the payoffs associated with various scenarios.

The next phase involves multiple periods of decision making in the adversarial setting. Each session lasts a minimum of 25 periods, after which each successive period occurs with a 75% chance, with a maximum of 35 periods. This end-game design creates uncertainty about which period is the last, thereby reducing concerns about idiosyncratic behaviors in the last period while still guaranteeing that the session will end within the announced time.

Out of the 16 total sessions, 14 were initialized to specific states for the first five periods of the experiment, in order to test how different initial conditions might alter the outcome of the experiment. To do this, the computer program randomly assigns certain specified proportions of subjects to each of the four strategies (Paladin, Apathetic, Informant, or Villain) in period one. During the first five periods, the subject's enforced choices are shown on the screen, and are not allowed to be altered by the subject. The subject is therefore aware of which strategy he or she is being forced to play for the initialization periods. The interactions and payoffs are recorded and shown to the subject during the initialization period. In the 2 remaining sessions with no initialization period, all subjects were allowed to choose their strategy in the first period. We employed three different initializations: 90% Villains - 10% Informants, 90% Villains - 10% Paladins, and 60% Villains - 40% Paladins. The first two start the system very near Dystopia to see the role of Informants in transitioning to Utopia. The third has a strong presence of Paladins but, under the deterministic imitation dynamic [26], does not converge to Utopia because Informants are missing.

Starting in the first period after initialization (period 6 in the 14 initialization sessions and period 2 in the no-initialization sessions), subjects are allowed to choose whatever strategy they would like (from the list of available strategies) at the beginning of each period, constrained only by the level 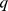 of behavioral inertia chosen for the experiment. This behavioral inertia randomly forces each subject to play the same strategy as played in the last period with probability 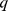, i.i.d. across subjects. We enforce a non-zero level of behavioral inertia to prevent rapid cycling between strategies and to make choices more salient to the subjects, as they are aware that whatever strategy they choose now may stick with them for an extended period of time. Most sessions used 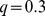, but we also ran two high inertia sessions with 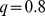 for robustness. As seen later, this change in inertia slowed convergence but played no other significant role.

**Figure 3:**
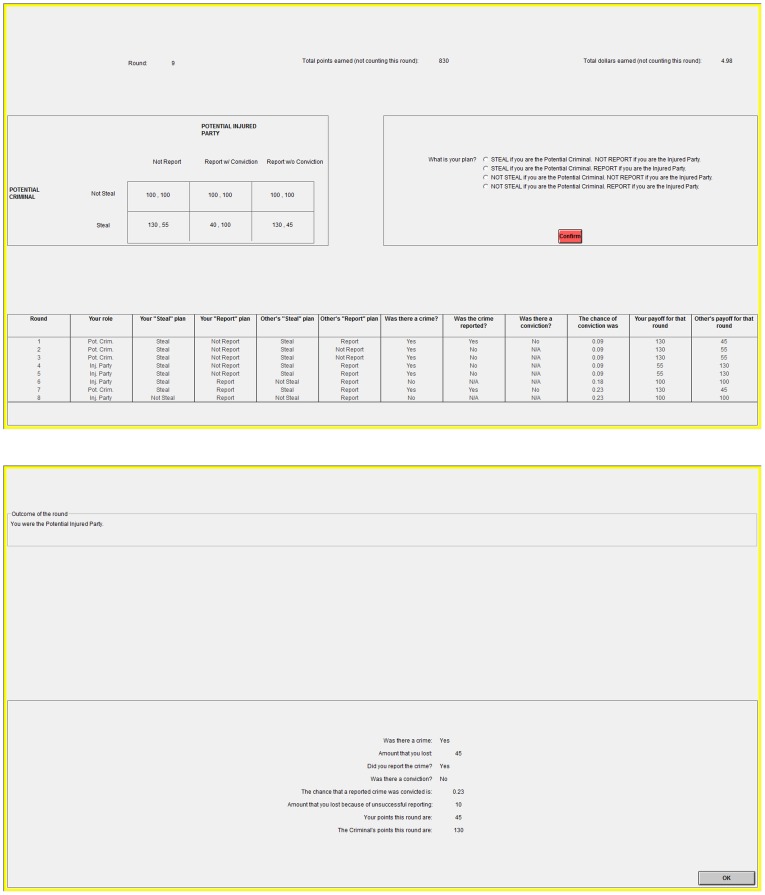
Screen captures of the decision screen (top) and results screen (bottom) in our experimental software.

Within each decision making period of the experiment, there is a decision screen and a results screen, screen shots of which are shown in Fig. 3. If a subject is allowed to select a strategy that period, he or she does so by clicking one of four options: “NOT STEAL if you are the Potential Criminal. REPORT if you are the Injured Party.”; “NOT STEAL if you are the Potential Criminal. NOT REPORT if you are the Injured Party.”; “STEAL if you are the Potential Criminal. REPORT if you are the Injured Party.”; or “STEAL if you are the Potential Criminal. NOT REPORT if you are the Injured Party.”. These strategies align with Paladin, Apathetic, Informant, and Villain, respectively. To prevent the creation of unintended focality, each subject is shown these options in a randomly determined order. This is done by randomly assigning to each player an “order type”, which determines the order in which the strategies are presented to that subject, at the beginning of the experiment. That is, one subject has the four strategies displayed in a certain order, another has a different order, and so on, but the order for a given subject remains fixed during the duration of the experiment to avoid confusion. Once a subject selects a strategy, he or she clicks a button labeled “OK” to signal that he or she is ready to move on to the results screen. Subjects who are not allowed to select a strategy for that period because of behavioral inertia (or during the initialization periods) are also required to click “OK” to signal that they are ready to advance to the results screen.

After each subject has selected “OK” to move on to the results screen, each participant is randomly and anonymously paired with another participant, and the program randomly assigns the roles of “potential criminal” (player one) and “potential injured party” (player two) within each pair. The program then plays out each player's strategy based on the assigned roles. Four possible outcomes can occur. First, if player one selected to not steal (Paladin or Apathetic), then both subjects receive their endowment of 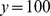 for that period. Second, if player one selected to steal (Villain or Informant) and player two selected to not report (Apathetic or Villain), then player one receives 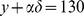 and player two receives 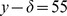 (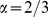, 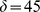 for both parameter profiles employed in our study). If player one selected to steal (Villain or Informant) and player two selected to report (Informant or Paladin), then the outcome is stochastically dependent on whether or not the criminal is convicted, which happens with probability 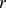, which is equal to the fraction of all players that chose to play as either an Informant or Paladin. Thus, the third potential outcome is that conviction occurs, wherein player one pays back 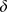 to player two and pays an additional cost 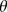, for a total payoff of 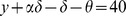 for player one (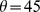 for both parameter profiles) and 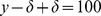 for player two. The fourth potential outcome is that no conviction occurs, wherein player one receives 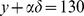 and player two incurs an extra cost of 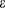, for a total payoff of 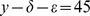 in parameter Profile A and 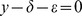 in parameter Profile B (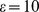 in Profile A and 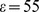 in Profile B). Note that the only difference between parameter Profiles A and B is in the additional retaliation cost for unsuccessful reporting, 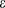. In expectation, it is thus more costly to report in Profile B than in A.

## Results

The top 5 entries in Table S2 provide information and outcomes of experimental sessions that are representative of the key patterns found across all 16 sessions; full results for each session are detailed below. When all strategies are allowed (as in Sessions 1 and 2), the subjects converge to Utopia, a result that is robust across all studied treatment conditions. Figure 2(B) shows the evolution of strategies from Session 1. Akin to what is observed in the simulation data in Fig. 2(A), we see a rise in Informants that precedes a rise in Paladins, which eventually leads to convergence at Utopia. Though this is cursory evidence that the dynamic effect Informants play in the experiments is similar to that predicted by the imitation dynamic theory, we provide a stronger test of this prediction by conducting sessions that disallow the Informant strategy. Indeed, in such sessions (as in 3 and 4), the system converges to Dystopia, a result that is, again, robust across all studied treatment conditions. Figure 2(D) shows the evolution of strategies from one such session, Session 3. To verify that convergence to Dystopia in these sessions is not due merely to a reduction in the strategy set, we examine Session 5, which excludes Apathetics. As suggested by the imitation dynamic theory, disallowing Informants prevents Utopia, but disallowing Apathetics does not.

The crucial role played by Informants vis-à-vis Utopia in the experimental sessions qualitatively matches the theoretical results, yet the data contain three striking patterns that reveal discrepancies with the theoretical work. First, Informants are vital for the emergence of Utopia under experiments performed with both parameter profiles, despite starting from initial conditions in which they should play no role under the theoretical best response dynamic [27]. This may be due to inherent behavioral proclivities of the subjects (e.g., a preference for reciprocity), which could cause them to at least partially discount the initial conditions forced upon them. Second, in Session 3, the subjects converge to Dystopia despite coming close to Utopia early in the session, as seen in Fig. 2(D). Here, 72% of the subjects are Paladins in periods 8 and 9, but the system thereafter experiences a rapid increase in criminal defection that remains in place for the duration of the session. The observed pattern suggests that Informants play a role not just in helping the system initially transition to Utopia, but also in keeping the system in Utopia once it is reached; this matches the role of Informants in the best response dynamic, but not in the imitation dynamic. Third, the majority-Villain Dystopia achieved in the experiments differs from those predicted by theory: in the experiments, the minority population are Paladins (see Fig. 2(D)), while under the imitation dynamics, the minority population are Apathetics (see Fig. 2(C)) and under best response there is no minority population. Again, this may be due to subjects' inherent preferences for certain strategies, namely the Paladin.

**Figure 4:**
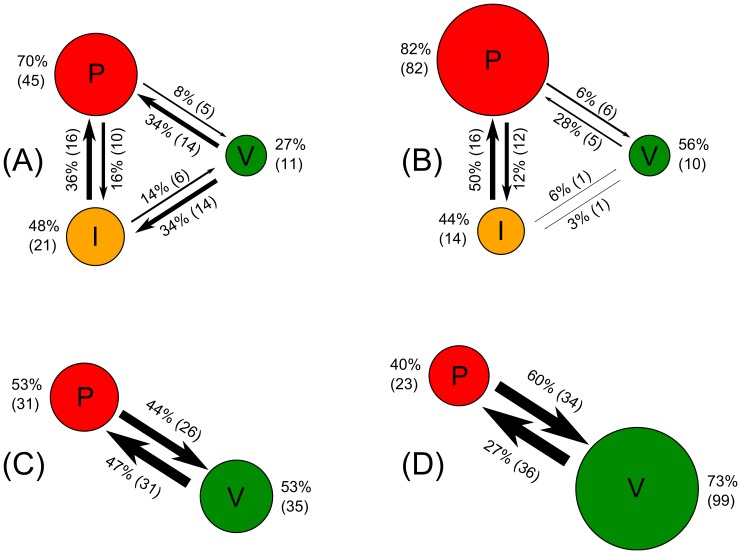
Strategy transition diagrams from Session 1 (top figures) and Session 3 (bottom figures), in both early (left figures) and late (right figures) periods. Apathetics have been ignored, as they played a negligible role. The sizes of arrows and circles represent the number of occasions in which subjects allowed to choose their strategy made the indicated transition or continued with their current strategy, respectively. These raw counts are indicated in parentheses near each arrow and circle, with accompanying percentages.

To help visualize the role Informants play in transitioning a population to Utopia and helping it remain there, we partition the data into early (1–15) and late (16-end) periods and calculate the number of instances in which a subject, when given the opportunity to switch strategy, either keeps his or her current strategy or chooses another. Figure 4(A) displays the observed transition distributions from the early periods of Session 1, ignoring Apathetics because they constitute a very small portion of the observed strategies. Here, we observe that Villains switch to Informants and Paladins at similar rates, but Informants preferentially transition to Paladins rather than to Villains. In effect, the Informant strategy provides a pathway by which Villains become Paladins during the transition from Dystopia to Utopia.

On the other hand, Fig. 4(B) displays the transition distributions from the late periods of Session 1. The system is in Utopia, but there is still noise and “mutation” as Paladins experiment with the Informant strategy before switching back to Paladins; this occurs far more frequently than the Paladin-Villain mutation. Observe that Informant mutants, although increasing the occurrence of crimes, still foster a high incidence of reporting, thereby sustaining the Paladin strategy as the best response, and reinforcing the Utopian state as the preferred equilibrium. Thus, Informants help the system maintain Utopia once it is reached.

The dramatic effect of removing the Informant strategy is evident in Figs. 4(C) and 4(D), which depict the transition distributions from the early and late periods of Session 3, respectively. In the absence of Informants, there is no robust pathway for Villains to transition to Paladins in the early periods of the game, while in the latter ones, if Utopia is achieved, the reinforcement provided by Informants is no longer present. Thus, the system is hindered in reaching Utopia and is unable to maintain any Utopian state achieved; Dystopia eventually prevails.

**Figure 5:**
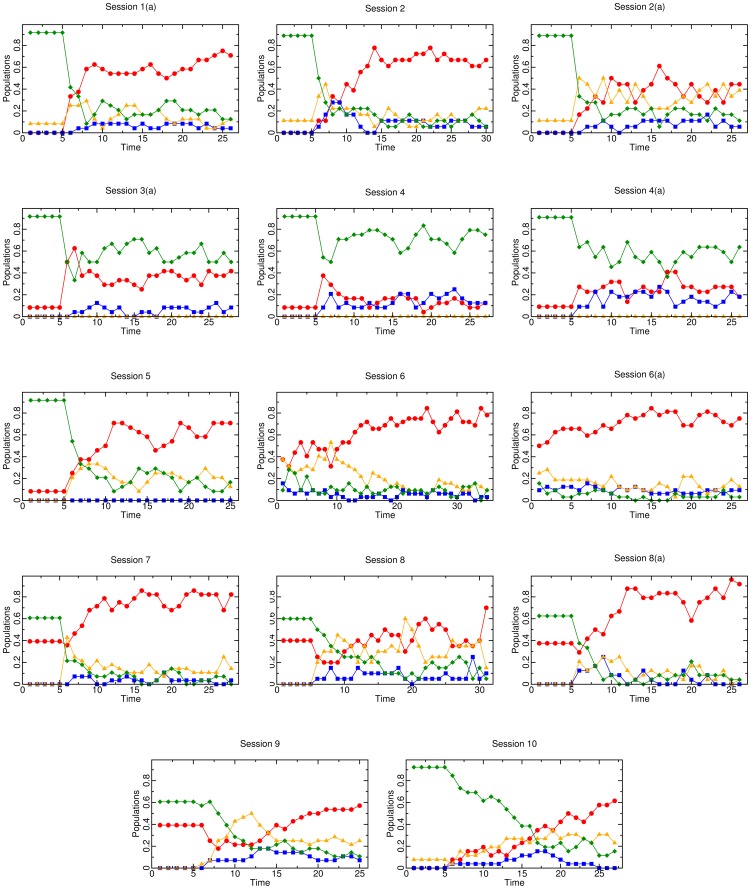
Strategy evolutions for 14 of the 16 experimental sessions. Results for the remaining two sessions are displayed in Fig. 2. In all cases, Paladins are red circles, Apathetics are blue squares, Informants are orange triangles, and Villains are green diamonds.

We now turn to a detailed look at all 16 sessions. Each session number corresponds to a unique treatment, while sessions with an “(a)” appended represent a replicated session of the same treatment number. We first present results obtained from sessions where all four actor strategies were available, then we consider those where Apathetics or Informants were forbidden, leaving only three available strategy choices. Each of these subsections is further subdivided into two parts, which describe the results for experiments run with Profile A and Profile B, respectively. We refer throughout to the evolution of strategy types presented in Figs. 2 and 5, along with the treatment details displayed and experimental results presented in Table S2.

### All Strategies Available

#### Parameter Profile A

Sessions 1, 1(a), 6, 6(a), 7, 9, and 10 all use Profile A, have all strategies available, and converge (or appear as though they will eventually converge) to Utopia. Sessions 1/1(a), 6/6(a), and 7 vary in how they are initialized, and Sessions 9 and 10 enforce a high level of behavioral inertia with different initializations.

Sessions 1 and 1(a) initialize the population at 90% Villains - 10% Informants for the first 5 periods and both exhibit a large proportion of Informants and Paladins present as soon as subjects are allowed to choose their strategies. As these sessions continue past these initial bursts, the Paladins bolster in numbers as the number of Informants declines. The trend of a rapid increase of Informants and Paladins in early periods followed by growing Paladins and dwindling Informants is seen in many sessions. Over the last five periods, Sessions 1 and 1(a) average at least 70% Paladins, thus ending in Utopia.

Sessions 6 and 6(a) are unique because they have no initialization periods. These are the only sessions where, in the first period, all of the subjects were allowed to select a strategy type. In both of these sessions, the Informant is the second most commonly played strategy (behind Paladin) for most periods. In Session 6, the number of Informants follows an upward trend from period 1 until period 9, where there are actually more Informants than Paladins (53% Informants versus 31% Paladins). After period 9, we observe the expected behavior of Paladins growing as subjects playing the Informant strategy declines. Over the last five periods, these two sessions average at least 75% Paladins and less than 10% Villains.

Session 7 initializes the subjects at 60% Villains - 40% Paladins, and behaves similarly to Sessions 6 and 6(a). Initial conditions were chosen closer to Utopia here than in Sessions 1 and 1(a), so it is no surprise that Session 7 ends in Utopia, and that there is an average of 79% Paladins over the last five periods.

Sessions 9 and 10 are unique because they enforce a very high level of inertia, 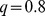, with initializations of 60% Villains - 40% Paladins and 90% Villains - 10% Informants, respectively. For the inertia value used, only 20% of the subjects are allowed to change their strategy each period after the 5 period initialization phase. We did this to check whether a high level of inertia would affect the ability of a population to reach Utopia. In Sessions 9 and 10, we only observe 54% of the subjects playing the Paladin strategy averaged over the last 5 periods. While this may seem low, the evolution of strategies pictured in Fig. 5 illustrates an upward trend in the number of Paladins in both sessions. Because of this, it seems likely that the population would have converged to a higher level of Paladins if it had been allowed to continue for more periods. Allowing only a small percentage of the subjects to change strategies each period provides a more fine-grained view of the role Informants play in the emergence of cooperation. This is because the high level of inertia allows us to better follow the early stage of the dynamics and to understand how Informants affect the behavior of a population near Dystopia. Both sessions show evidence of Informants “leading” the burst of Paladins, but this is more clearly shown in Session 9. In both sessions, from period 13 until the end of the session, the percentage of Informants is between 21% and 50%.

#### Parameter Profile B

Sessions 2, 2(a), 8, and 8(a) all use Profile B, have all strategies available, and converge toward Utopia. Sessions 2 and 2(a) are initialized at 90% Villains - 10% Informants for the first five periods. Under Profile B, the best response dynamic suggests that this low initial level of Informants should prevent the transition to Utopia, but the evolution figures for Session 2 and 2(a) again illustrate the Informant strategy “leading” the Paladin strategy. Within the first two periods after the initialization period, the percentage of Informants is up to 44% and 50% in Session 2 and 2(a), respectively. In Session 2, the percentage of Paladins has spiked at 78% by period 14. The percentage of Paladins hovers right at, or slightly below, this number for the remainder of the experiment. Session 2(a) did not quickly adopt such a high percentage of Paladins, but we conclude that Session 2(a) was close to converging to Utopia. This is mainly because of this session's consistently high conviction rate, 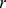. By period 6, Session 2(a) has a conviction rate of 67%, and 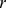 stays above this number for the remainder of the session. With such a high conviction rate, it is likely that more periods of play would have led to most non-Paladin strategies switching to non-stealing strategies, most likely Paladins.

Sessions 8 and 8(a) are the same as Sessions 2 and 2(a), except that they are initialized for the first five periods with 60% Villains - 40% Paladins. Since we observed Sessions 2 and 2(a) converge to Utopia, we would expect that an initialization *closer* to Utopia would produce the same result. This is what we observe from Sessions 8 and 8(a). Session 8 exhibits similar behavior as Session 2(a), in that, while the system only has an average of 44% Paladins over the last five periods, the reporting rate is very high: 76% over the last five periods. If more periods had been played, it is likely that we would have observed subjects switching to non-stealing strategies, most likely Paladins. Session 8(a) displays a steady rise in Paladins and an average reporting rate of 91% over the last five periods.

### Three Strategies Available

#### Parameter Profile A

Sessions 3 and 3(a) do not allow the Informant strategy, and are of primary interest because they are the only sessions under parameter Profile A that do not converge (or are not on a clear path to converge) to Utopia. Both sessions are initialized near Dystopia (90% Villains - 10% Paladins) for the first five periods and average over 55% Villains over the last five periods. In comparison, the highest percentage of Villains in any of the 12 sessions that converge to Utopia is 18% (Session 9). The evolution of the strategy types gives credence to the theory that Informants play a role in stabilizing Utopia. The lack of a pro-reporting criminal type (Informant) causes instability for the players in coordinating on non-criminal behavior. This is seen in both sessions, but more clearly in Session 3, where the percentage of Paladins jumps up to 72% in periods 8 and 9 before that number plummets down to 27% in period 13 and down to 6% in period 20. Session 3(a) displays similar behavior with a jump of Paladins to 63% in period 7 and a sharp decline in the next period to 38%. The proportion of Paladins in Session 3(a) hovers around 33% for the remaining periods. Without the Informant strategy available to stabilize the initial burst of Paladins, the number of Paladins falls and the number of Villains rises.

Session 5 is the only treatment that restricts the set of possible strategies while still including the Informant strategy. This treatment was used to test if the Apathetic strategy played an analogous role to that of the Informant. The Apathetic and Informant strategies are theoretically analogous, in that they are both hybrid strategies that mix defection and cooperation. This theoretical similarity is not illustrated in the experimental results. The fundamental difference between the Informant and Apathetic strategies is clearly seen when examining the frequency at which each of these strategies were chosen in the experiments. When only counting the periods where subjects were allowed to choose strategies (not in the initialization periods or restricted by behavioral inertia), the Informant strategy was chosen much more often than the Apathetic strategy. In the 15 sessions when the Apathetic strategy was available (all sessions other than Session 5), subjects only selected it 7.6% of the time. Additionally, there were 53 periods out of a possible 343 where no one in the session was playing the Apathetic strategy even though it was available. Comparatively, in the 12 sessions when the Informant strategy was available, subjects selected it 21.4% of the time, and there was only one observed period out of 270 where no one selected the Informant strategy. This happened in Session 8(a) on period 25, where there were 96% Paladins. In Session 5, we observe similar results to other treatments that *do* allow the Apathetic strategy: an initial boom of Informants and Paladins, followed by a continuing upward trend of Paladins with a downward trend of Informants. Session 5 provides evidence that the Informant and Apathetic strategies are not analogous hybrid strategies to one another, and that, indeed, there is something unique about the Informant strategy.

#### Parameter Profile B

Sessions 4 and 4(a) both initialize the population at 90% Villains - 10% Paladins, do not allow the Informant strategy, and converge to Dystopia. These sessions are crucial in illustrating the vital role of the Informant in leading to the emergence to cooperation. While there is an initial “burst” of Paladins in the first periods after initialization, it is not as dramatic as the burst in Sessions 3 and 3(a). In period 6, Sessions 4 and 4(a) experience a jump in Paladins to 38% and 27%, respectively. Both sessions experience a dwindling or stagnation in the percentage of Paladins after this period for the rest of the session. Session 4(a) exhibits another small burst of Paladins in periods 17 and 18 at 41%, but on average, both sessions report low numbers of Paladins toward the end of the session. Over the last five periods of Session 4, only 12% of subjects are playing the Paladin strategy compared to 73% choosing the Villain strategy. Over the last five periods of Session 4(a), only 25% of subjects are playing the Paladin strategy, and 60% are playing the Villain strategy.

## Discussion

To summarize, we find experimental evidence that the Informant strategy, which allows actors to defect by committing crimes but cooperate by assisting in the punishment of other criminals, is critical for the emergence of overall cooperation in an adversarial setting. In our experiments, Utopia is always converged upon when the Informant strategy is available and never converged upon when it is not, regardless of parameter profiles. The Informant strategy is also found to be essential for keeping the system in Utopia once it is reached. Overall, our results constitute new evidence that transitioning from a high crime to low crime society may require successful cultivation of actors that both commit crimes and cooperate with authorities.

It is instructive to compare our findings with other research on the emergence of cooperation. The robustness of our findings is supported by prior theoretical work [31], [32] on spatial public goods games containing four strategies analogous to those considered here, in which “punishing defectors” (the analog of Informants) can help stabilize cooperative states, though only under very small regions of parameter space. Unlike the commonly studied social dilemma settings with punishment, the adversarial setting we study does not manifest a second-order free-rider problem in which the decision to punish or not is in itself a social dilemma. Because the conviction probability 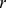 is increasing in the number of reporters, the second-order punishment game is in fact a coordination game where the expected cost of reporting vanishes as the proportion of reporters approaches one, i.e., as the system approaches Utopia [27]. Consequently, our adversarial setting is more similar to works in which coordination fosters punishment [2], [33], than to those in which the cost of punishment is direct and independent of the punishment decisions of others [34]. Our results thus complement prior evidence that coordinated punishments can help both the emergence and sustenance of cooperation, albeit in a newly studied, directly adversarial setting.

## Supporting Information

Table S1 **Subject details.**
(TIF)

Table S2 **Session details and outcomes.**
(TIF)
