# Supplementary figures and images for: Criminal Defectors Lead to the Emergence of Cooperation in an Experimental, Adversarial Game
Source: PLoS One. 2013 Apr 23;8(4):e61458. doi: 10.1371/journal.pone.0061458 (PMC3634082; doi:10.1371/journal.pone.0061458)

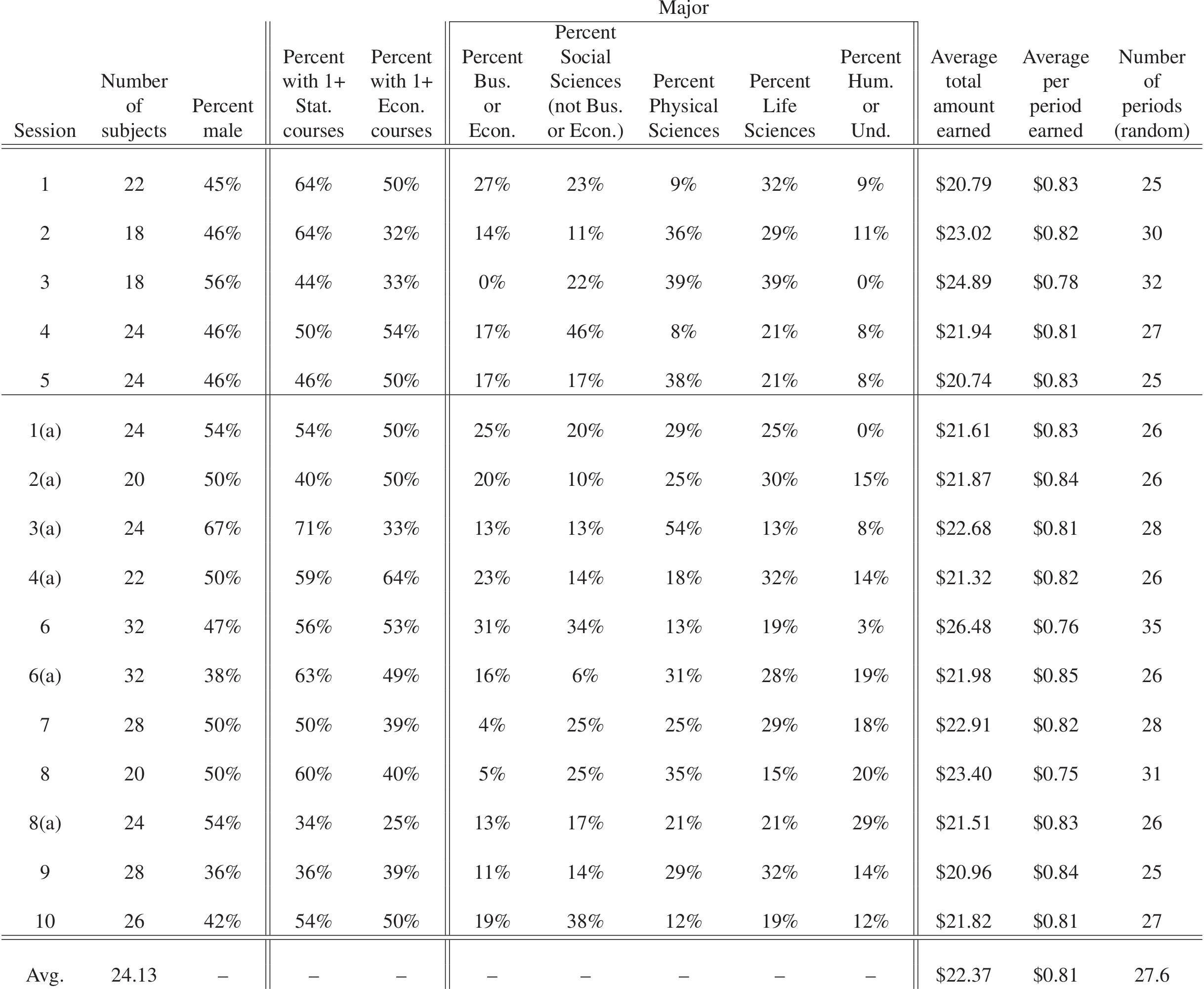

Supplement: Table S1 — Subject details. (TIF) [file pone.0061458.s001.tif]

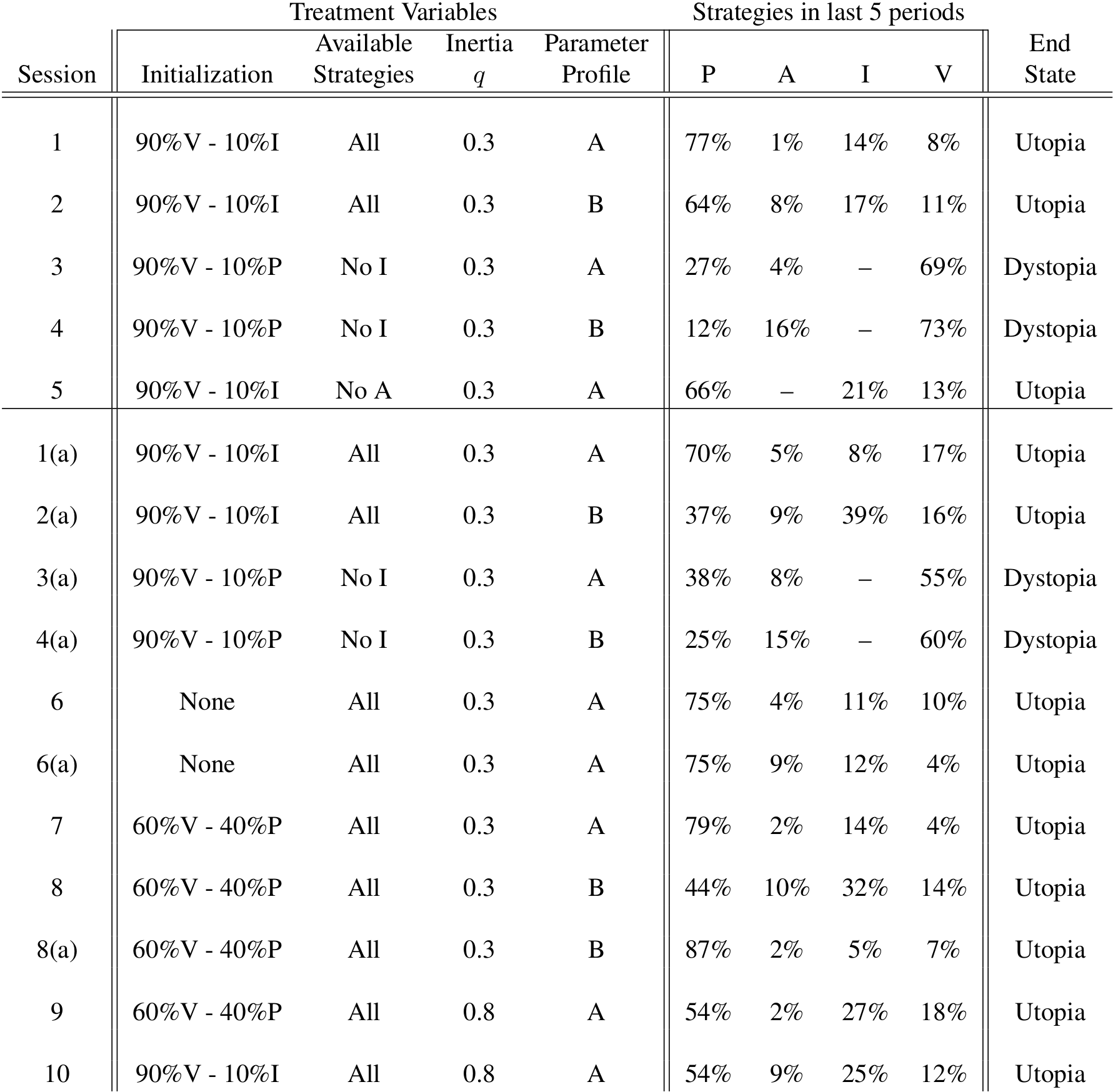

Supplement: Table S2 — Session details and outcomes. (TIF) [file pone.0061458.s002.tif]
